# Vitamins and other immune-supportive elements as cofactors for passing the COVID-19 pandemic

**DOI:** 10.1186/s43088-021-00163-2

**Published:** 2021-10-29

**Authors:** Haitham Saeed, Hasnaa Osama, Mona A. Abdelrahman, Yasmin M. Madney, Hadeer S. Harb, Mohamed E. A. Abdelrahim, Fatma Ali

**Affiliations:** 1 Clinical Pharmacy Department, Faculty of Pharmacy, Beni-Suef University, Beni-Suef, Egypt; 2 Food Hygiene, Department, Faculty of Veterinary Medicine, Beni-Suef University, Beni-Suef, Egypt

**Keywords:** COVID-19, Coronavirus, Nutrition, Vitamins, Immunity, Traced elements

## Abstract

**Background:**

Coronavirus disease 2019 (COVID-19) is a viral disease that causes a respiratory disorder, started in December of 2019 in China. Several vitamins and trace elements could help in enhancing host immunity producing antioxidant or anti-inflammatory action. This work aimed to identify the role of different nutrition, vitamins, and trace elements on the immunity status of the infected subject and the possibility of the beneficial role of these elements in the management of COVID-19.

**Main body:**

After collecting (PubMed, scholar, OVID, Embase, Cochrane Library) and investigating published articles, testing the effect of these elements on viral infection, it was found that most of these elements have a significant role during viral infection through a different mechanism, like antioxidant, anti-inflammatory, and immunomodulation. Nutritional interventions in COVID-19 infections are very important currently, and it was reported that vitamin C and D reduce the risk of acute respiratory infections. In addition, low vitamin A diets compromise the effectiveness of inactivated bovine coronavirus vaccines. Administration of N-acetyl cysteine showed a beneficial inhibitory effect in viral infections and enhanced glutathione production. The deficiency of selenium on COVID-19 subjects has a significant impact on the clinical outcome of the subjects. In addition, supplementation with vitamins proved to enhance immune response during viral infection. Vitamins and trace elements not only showed a beneficial effect but also Omega 3 fatty acids showed an immunomodulating effect during infections.

**Short conclusions:**

Assessment of levels for these trace elements at the baseline and providing supplementation containing different vitamins and elements could result in better control and clinical outcomes in the case of COVID-19.

## Background

The new coronavirus disease started in China in December of 2019 and has become a worldwide pandemic disease with millions of confirmed cases. COVID-19 is a respiratory disorder that is presented by flu-like symptoms as fever, dry cough, and fatigue. The severity of the COVID-19 ranges from mild cases that represent more than 80% of confirmed cases to severe cases with respiratory limitations that need for intensive care unit support. Sever COVID-19 cases are mainly old age patients or those with comorbidities [1, 2].

In 1970, the relationship between the disciplines of immunology and nutrition was completely established and reflected by using immunological measures as a component for the assessment of nutritional status [3, 4]. The COVID-19 pandemic is causing severe threats to international health and the economy. At the moment, there are still no proven therapeutic agents for the disease and more alternative methods need to be found to control the virus's spread and effect. Nutrition may have a positive impact on the prognosis of COVID-19, 22% of deaths among adults in 2017 were as a result of poor diet, and healthy patterns of eating enhance the function of the immune system and also improve immune metabolism [5].

Malnutrition is a predictor of the prognostic direction for patients with respiratory failure and those on mechanical ventilation. Malnutrition could be a result of different deficiencies; one of them is low albumin level, which is associated with the case prognosis [6].

Protein synthesis and division of immune cells require a high amount of amino acids. Hence, protein-energy malnutrition (PEM) is considered the main reason behind immunodeficiency worldwide [7–9].

Not only proteins have a role in building immunity against infections but also adequate amounts of elements like fatty acids, and vitamins have a role. Vitamins that could influence the immune status are vitamins A, C, D, and E. Essential elements that play an important role in enhancing immunity are iron, selenium, and zinc [9–11].

Recently, a few studies recommended the evaluation of the nutritional status of COVID-19 subjects at the time of admission to enable the health care workers from balancing and normalizing the nutritional requirement of the case [12, 13]. Subjects that are malnourished should receive nutritional supplementation as early as possible, especially increasing oral intake of amino acids [12]. An adequate protein intake of 1.5 g/d should be maintained for all COVID-19 cases even they are not presenting to the hospital with signs of malnutrition [12]. Antioxidant and anti-inflammatory properties of some vitamins and nutrients could be a reason behind their involvement in the nutritional plan of the COVID-19 subjects [13].

This article aimed to review and identify the role of several nutrients in maintaining host immune defense against COVID-19.

## Main text

### Amino acids

In cellular proteins, glutathione acts as a barrier against reactive oxygen species (ROS) produced by several viral infections including COVID-19, but excessive production of ROS results in depletion of the defending proteins and worsening of COVID-19 patient’s condition [14–16]. A recent study indicated that the glutathione, glutathione peroxidase activity was lower in COVID-19 patients compared to the control group as a result of increased oxidative stress [16]. Glutathione is the main defensive antioxidant synthesized from three amino acids involving cysteine, glycine, and glutamic acid. It acts as a barrier that interacts with ROS before damaging the cellular component [14]. The depletion of glutathione and other antioxidants leads to more viral spread and replication by providing a more convenient environment [17–19]. Hence, providing the main amino acids for producing glutathione is beneficial in enhancing the immune response in the case of viral infections. Administration of N-acetyl cysteine resulted in enhanced cell-mediated immunity also decreased the severity of the flu-like symptoms and showed beneficial clinical outcomes for COVID-19 patients [20, 21]. In addition, the effect of N-acetyl cysteine was also demonstrated on the coronavirus infection in animals that cause diarrhea and showed a beneficial effect in reducing the severity of cell injury [22].

Clinical studies showed a beneficial impact of using N-acetyl cysteine and glutathione for the management of COVID-19 patients regarding discharge rate, oxygen therapy duration, and mortality rate [21, 23–25]. Hence, focusing on nutritional support and supplementation with cysteine during the COVID-19 pandemic could lead to higher control and a good prognosis [26].

### Dietary long-chain polyunsaturated fatty acid

Fatty acids are important mediators that are involved in the immune response, especially omega-3 dietary long-chain polyunsaturated fatty acids (n-3 PUFAs) [27]. PUFA which is derived from fish oil was indicated to enhance several autoimmune and chronic inflammatory disorders [28]. Docosahexaenoic acid (DHA) and eicosapentaenoic acid (EPA) were the most effective acids that have a beneficial effect on the immune system by decreasing the severity of diseases; however, most supplementation containing PUFA showed a favorable immunological effect [29, 30]. The mechanism by which PUFA could act on the immune system during infections is by regulating several inflammatory processes. Hence, n-3 PUFAs could play an important role in reducing COVID-19 inflammation due to having anti-inflammatory properties [31, 32]. The anti-inflammatory effect of n-3 PUFAs is related to certain active metabolites such as protectins and resolvins. The anti-inflammatory action of n-3 PUFAs results from the ability to prevent translocation of nuclear p65 NF- κB which leads to decreased activation of NF-κB. Also, n-3 PUFAs have another anti-inflammatory action related to the reduction of Cyclooxygenase 2 (COX-2) [33]. In addition to the anti-inflammatory effect, the antioxidant effect of nutritional supplements could improve the clinical outcome and oxygen saturation of critically ill patients during intensive care unit stay, particularly in patients suffering from acute respiratory distress [34, 35]. However, the role of omega-3 supplementation in the management of acute respiratory distress syndrome (ARDS) should be better clarified by more studies and clinical trials, but also its vital role in decreasing reactive oxygen species and pro-inflammatory cytokines, such as IL-1β, IL-6, and IL-8 [36], is well clarified.

Patient with pneumonia or COVID-19 has limited lung functions resulting from excessive inflammation that could be improved by the administration of PUFA [37, 38]. The up-regulation of specific and nonspecific immune defenses of the host is considered the main way by which n-3 PUFA could exert its beneficial effect during acute pneumonia [39]. Fish oil is ever more consumed by the public during the COVID-19 pandemic [40] due to its potential medical benefits, it could be used for enhancing the management of several diseases including COVID-19, and also, it could be helpful for survivors [41, 42]. Some studies have indicated that n-3 PUFAs have immunosuppressive and immunoregulatory properties so it may increase the risk of the subject being infected; however, there are no studies that indicated such effect in COVID-19 patients [29]. Unlike animal studies, some human studies tested the effect of fish oil supplementation on the immune system showed no immunological effect [43, 44]. Hence, the administration of n-3 PUFA supplementation could be advantageous for some and non-beneficial for other people; therefore, it should be administrated with caution. However, the administration of n-3 PUFA as a dietary supplement like sardine, salmon, canola, and soy is recommended [29].

### Vitamins

#### Vitamin A

Vitamin A is a fat-soluble vitamin that could be present in several fruits and vegetables such as orange and yellow vegetables and fruits, and also, it could be found in other sources like eggs, cod liver oil, fortified skim milk. Vitamin A has a generally beneficial effect on the human body like maintaining and promoting mucosal integrity, promoting growth, and maintaining vision, and also, it has a specific role in enhancing host immunity and regulation of hormonal responses [45]. Hence, deficiency of vitamin A is considered a big issue due to its role in enhancing immunity and fighting against microbes particularly in developed countries. The level of vitamin A was significantly lower for COVID-19 patients suffering from acute inflammation, ARDS, compared with control [46]. The deficiency of vitamin A is mainly related to PEM because it is mainly obtained from animal sources like poultry, dairy product and, meat, so management of vitamin A deficiency could be achieved through treatment of PEM and consumption of a high protein diet [47, 48]. Also, the administration of vitamin A has beneficial effects in COVID-19 patients, and it has anti-inflammatory properties, enhances immunity, and plays a role against oxidative stress [49].

Administration of vitamin A (200,000 I.U.), two doses for two days, showed a significant improvement in COVID-19 symptoms, duration of illness, deterioration rate compared with the control group [50]. Besides, the administration of vitamin A to contacts of COVID-19 patients also showed a beneficial effect related to the incidence of infection [50].

#### Vitamin C

Vitamin C is a water-soluble vitamin that is commonly used during respiratory infections due to its ability to reduce the severity and duration of the disease by enhancing the immunity of the host [51]. During infection, vitamin C which presents in leukocytes is rapidly used up [29]. Vitamin C has antioxidant properties and also acts as an enzymatic cofactor for several physiological reactions, such as enhancing the host immune system [52]. Administration of IV vitamin C for COVID-19 patients resulted in a substantial reduction in inflammatory markers, including D-dimer and ferritin, and decreased oxygen requirements [53]. In addition, Kumari et al. indicated a significant beneficial effect of IV vitamin C in recovery from COVID-19 symptoms and hospital stay, but without significant impact on mortality rate or need for ventilation [54].

A multicenter study was carried on COVID-19 patients with severe cases, they received high dose IV vitamin C for 7 days, and the outcomes that had been measured were patient's need for ventilation, mortality rate, oxygen status, and inflammation markers. Administration of vitamin C resulted in significant anti-inflammatory effect through reduction of IL-6 and the oxygen levels of patients were improved while ventilation and mortality rate showed non-significant difference [55]. On the other hand, other trials indicated no significant differences were related to adding vitamin C to the traditional regimen for COVID-19 subjects [56, 57].

Several observational studies were done for evaluation of the vitamin C role in the management of COVID-19. These studies showed a beneficial impact for using vitamin C in relieving symptoms, hospital stay, and increasing survival rate [54, 58]. Another role of vitamin C besides its ability to enhance host immunity is the minor antihistaminic effect which could aid in providing symptomatic treatment of the flu-like symptoms such as swollen sinus and sneezing [3, 59, 60].

A recent meta-analysis that analyzed results of eight clinical trials indicated a significant beneficial impact of vitamin C on the length of mechanical ventilation by about a 14% reduction rate [61].

COVID-19 is a result of the presence of lower respiratory tract infection; hence, providing vitamin C to the patient is recommended particularly through the dietary intake of vitamin C sources such as fruits and vegetables [62].

#### Vitamin D

Vitamin D is highly recommended by several studies to be involved in the process of COVID-19 management [63–65]. This recommendation is based on the different mechanisms of vitamin D that might improve the immunity status of the patient [66–68]. Vitamin D has many roles in enhancing immunity [68, 69]. It has a proven beneficial effect related to three different mechanisms: Cellular natural immunity, antioxidant, and adaptive immunity [64, 68, 70]. There is considerable evidence to show that vitamin D insufficiency is linked to COVID-19 severity and mortality, severity of COVID-19 disease increases as the level of vitamin D decreased [71–73].

The action of vitamin D on innate immunity is related to increased expression of defensins, human cathelicidin, and LL-37 that are antimicrobial peptides acting against the invasion of microorganisms [74, 75]. These natural internal peptides cause an upsetting of the infecting microbes membranes, and also, they could act differently through reducing microorganism endotoxins activity [76]. Some of the antimicrobial peptides such as cathelicidin has broad-spectrum activity against a broad range of bacteria, fungi, and viruses, and also, it could act against enveloped and nonenveloped viruses [77]. Besides, anti-inflammatory cytokines like IL-10 are upregulated by vitamin D, whereas pro-inflammatory cytokines like IL-1, IL-6, and tumor necrosis factor-alpha are downregulated. The likelihood of a cytokine storm in COVID-19 could be reduced by switching from a pro-inflammatory to anti-inflammatory condition [78].

The previous meta-analysis by Pal et al. has identified 13 studies that investigated the role of vitamin D administration before and after the diagnosis of COVID-19 symptoms [75]. The finding of the study reflected the significant role of vitamin D that had been administered after diagnosis of COVID-19 on Intensive care unit (ICU) admission and mortality rate while there was no significant beneficial impact on clinical outcomes or prevention of infection for patients who received vitamin D before the diagnosis of COVID-19 [75].

Viruses attach human cells and disrupt the integrity of the junction to facilitate the entrance of the virus, and this action is opposed by the ability of vitamin D to maintain a tight junction and act as a supporter of the physical barrier [79–81]. In a recent single-center, retrospective cohort study, it was indicated that low vitamin D levels were linked to a higher risk of severe COVID-19, implying that randomized trials are required to assess whether vitamin D affects COVID-19 severity [82]. Another study found that the majority of critically ill COVID-19 ICU patients with low vitamin C and vitamin D serum levels proved to be co-dependent risk factors for mortality [83].

#### Vitamin E

Vitamin E is one of the fat-soluble vitamins which is available in meat, cheese, shellfish, grains, and cereals [84]. It has an antioxidant effect besides its ability to modulate immune responses of the host, and also, its deficiency is related to enhanced viral antigenicity and decreased immune responses [84–87]. Besides, lower levels of vitamin E in COVID-19 subjects could be related to the poor prognosis of the disease through enhanced oxidative stress [88]. A previous study indicated that most COVID-19 patients suffer from micronutrients such as Vitamin D and E; the deficiency of vitamin E was noted in about 7% of patients, while it was near half regarding vitamin D [89].

In vivo studies indicated the beneficial role during viral infections, and its role was expressed by the anti-inflammatory effect and decreasing viral load [90]. However, in contrast to its beneficial effect, other studies have indicated that supplementation of vitamin E might result in harmful or no significant effects during the incidence of infectious disease. Regarding the non-significant effect of vitamin E, a previous study by Medydani et al. showed that the administration of 200 IU of vitamin E per day did not result in a significant effect on included subjects [91]. There are no sufficient studies regarding the use of vitamin E in COVID-19 cases, while some studies linked its deficiency to the increased severity of the disease. Its expected beneficial effect is mainly related to the antioxidant and immunomodulating effect.

### Traced elements

#### Selenium

Selenium is an important trace element that has antioxidant effects and anti-inflammatory properties [92]. Deficiency of selenium during animal studies showed that animals were unable to produce sufficient amounts of selenoproteins which is antioxidant and that leading to increased severity of the infection by allowing more virulent mutation of the virus [93, 94]. These findings were consistent with that of COVID-19 patients, and it was found that the selenium level is directly related to clinical outcomes and disease severity [95].

Levels of selenium and selenium transporter (SELENOP) for COVID-19 patients were lower than normal as reported by Moghaddam et al. Besides levels of selenium were associated with the disease severity, selenium was significantly lower for non-survived COVID-19 patients compared with less severe survived cases [96].

Several studies demonstrated evidence of the antiviral effects of selenium [97–99]. There are multiple mechanisms by which selenium could influence the viral pathogenicity, involving selenium-dependent glutathione peroxidases [99], thus would lead to the oxidative stress associated with several viral infections [97–99]. A recent study showed a significant link between selenium deficiency and the poor clinical outcomes of the COVID-19 subjects [92].

The role of selenium in fighting viral infection has been well-documented with many previously mentioned studies, and also, its deficiency effect on clinical outcomes of COVID-19 subjects [92, 97, 99, 100]. Hence, assessment of selenium status at baseline for COVID-19 subjects could be beneficial for more controlled management and better outcomes.

#### Zinc

Zinc is an important dietary trace element available in seafood, red meat, and poultry. It has immunomodulating properties related to both innate and acquired responses to viruses, and also, it plays an essential role in growth and development [101, 102]. The deficiency of zinc has been related to the increased severity of COVID-19 cases, and its deficiency was associated with reduced lymphocytic count [16]. Hence, the patient’s zinc status showed be assessed at baseline as it is an essential factor that could affect the host immunity toward COVID-19 infections [16]. A short recovery time for COVID-19 patients who receive high dose oral zinc supplementation was recently reported [103].

Direct inhibition of viral replication, improved mucociliary clearance of SARSCov2, improved pulmonary and renal tissue healing after ischemia, reduction of secondary bacterial infection possibility, restoration of interferon-alpha production, and immunomodulation are some of the mechanisms by which zinc may help COVID19 patients [104, 105].

Regarding the effect of zin supplementation on the clinical outcomes of respiratory diseases, a previous study carried on pediatric subjects with pneumonia that were receiving zinc showed that the group of pediatrics that received zinc has a significantly improved outcome [106]. Finally, zinc supplementation for vulnerable groups is also recommended through the administration of adequate amounts of dietary zinc sources [107, 108].

## Conclusions

Malnutrition is considered a big drawback against well-controlled management of viral infections, and these findings were confirmed from many studies that showed the effect of deficiency of vitamins (A, C, D, E) and trace elements (Zinc, Selenium) on the prognosis and clinical outcome in case of COVID-19. The role of Vitamin D, Vitamin C, and selenium in the case of COVID-19 is critical. Also, N-acetylcysteine supplementation showed a beneficial inhibitory effect on virus replication and enhanced glutathione production for Covid-19 patients. Hence, assessment of levels for these elements (Selenium and Zinc) and vitamins at baseline and providing supplementations containing different vitamins, nutrients, and trace elements could result in better control and clinical outcomes in the case of COVID-19. Providing these vitamins and elements to healthy subjects showed no beneficial impact, while its significant impact was reported for diagnosed COVID-19 subjects. Large multicenter trials are still needed to ensure the role of each element. The role of omega 3 is controversial, and there is no strong evidence for its inclusion as an adjunct therapy for COVID-19 subjects. These vitamins and elements should not be used alone to fight against COVID-19 but to be used as adjunct therapy with the traditional drug therapy.

## Data Availability

The datasets analyzed during the current study are available from the corresponding author on reasonable request.

## Abbreviations

ARDS: acute respiratory distress syndrome
COX-2: cyclooxygenase 2
COVID-19: coronavirus disease 2019
DHA: docosahexaenoic acid
EPA: eicosapentaenoic acid
HIV: human immunodeficiency virus
ICU: intensive care unit
n-3 PUFAs: omega-3 dietary long-chain polyunsaturated fatty acids
PEM: protein-energy malnutrition
